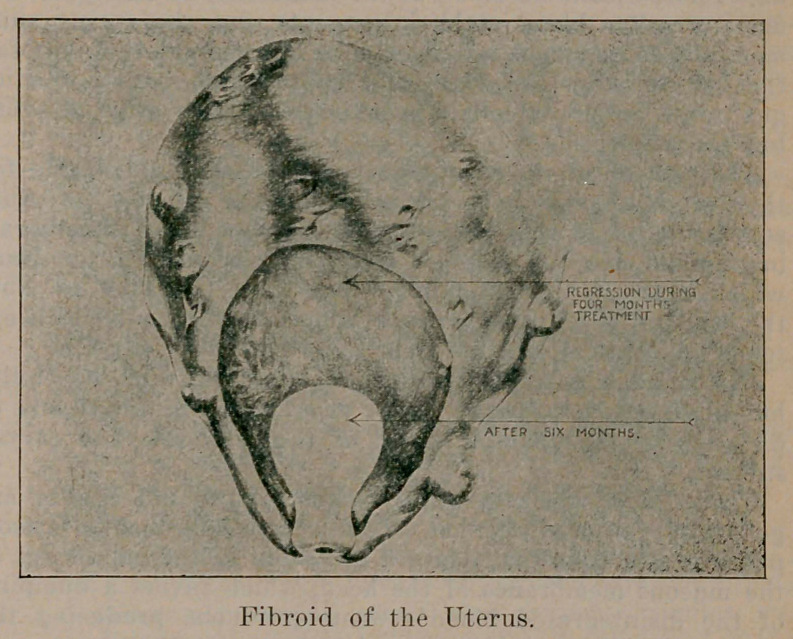# Radium as a Therapeutic Agent

**Published:** 1918-01

**Authors:** John M. Lee

**Affiliations:** Rochester, N. Y.


### BUFFALO MEDICAL JOURNAL

Yearly Volume 73 JANUARY, 1918 Number 6

### ORIGINAL ARTICLES

The right is reserved to decline papers not dealing with practical medical and surgical subjects, and such as might offend or fail to interest readers. Contributors are solely responsible for opinions, methods of expression and revision of proof.

### Radium as a Therapeutic Agent.

By JOHN M. LEE, M. D., Rochester, N. Y.

When surgeons were convinced many years ago that inflammation of the bowels and peritonitis caused thousands of deaths preventable by timely removal of the appendix, you remember how bitterly that operation was opposed for a number of years.

In the early days of abdominal section for the cure of ovarian tumor, a prominent French surgeon in referring to some of Dr. McDowell's reports says, "In spite of all that has been written respecting the cruel operation, we entirely disbelieve that it ever has been or ever will be performed with success." The French surgeon but expressed the prevailing sentiment of that time concerning Dr. McDowell's operation which has added ten years to the life of women. This opposition has been the rule with most advancements, whether among the laymen or the profession. In medicine and surgery, there have been gallant leaders who attacked what later even they acknowledged to be the best method of treatment.

The effects of radium upon such deadly disease as cancer are so startling that it is no wonder that men who have not had the opportunity to investigate the subject believe that it is another of the numerous fake cures for the red plague, and that it is only a kindness to warn people against submission of their cases to these over-enthusiastic, if not misguided doctors.

Comparatively, it is only a few years since the discovery of radium; its use in surgery is still more recent; and why should it not also receive persecution and condemnation?

This great advancement in cancer therapy ranks with anaesthesia antisepsis or any other of the great epoch making developments in surgery, and, like these it has had its months and years of earnest discussion. During this process of winnowing out the chaff, it is no wonder that the use of the agent was largely laid aside for a time because the methods of application were not sufficiently well understood to enable physicians to secure the desired results. Then, again, many of the specimens employed by the physicians were not up to the present standard. In those days, there was no standard, and in some instances where it was supposed that a goodly amount of radium was purchased, only a few milligrams were actually obtained. Very likely the instruments for weighing were imperfect and the mistakes were honest ones.

Nevertheless, the effect on surgical practice was uncertain, inefficient, and, of course, failed to produce the results expected because there wasn't sufficient dosage of the precious metal to effect cures. The writer has been told by one of the authorities in an American Company, that in weighing up a supposed fifty milligram tube, they found that there was less than five milligrams of the pure radium element contained in the receptacle. In another instance as the part of an estate of a physician which went into the hands of a receiver a twenty-five milligram tube of radium had to be measured and it yielded less than a single milligram of the radium element. The doctors, who made the purchases in these particular instances, not only lost several thousand dollars in actual cash, but, what was of even greater value to them, their reputations. These are some of the conditions which brought radium into disrepute in America. Up to this time, the people thought well of it, and many physicians caused their patients to rent tubes of the metal for use in malignant diseases. The writer recalls one particular case where a gentleman had cancer of the tongue; he paid $250 rental for the use of a tube for a week and carried it in his vest pocket when he did not hold it upon his tongue, which he was advised to do for only a few minutes at a time. There was no perceptible effect upon the disease at the end of the week from application of the radium and the carrying of the supposed metal in the pocket most of the time had no effect upon the skin, or tissues adjacent to the radium. This very fact that he could keep it in his vest pocket most of the time without any reaction of the skin underneath the pocket is proof conclusive that he really had but little, if any, radium in the tube. There were numerous cases of rental of radium over the country and the results were universally bad, and the explanation is the deception as to the amount of radium used. There need be no doubt as to the amount of element contained in any applicator or tube today as the contents of the apparatus may be checked up by the Bureau of Standards at Washington, D. C.

Another cause was the purchase and use of radio active materials which contained .no radium element and their activity lasted only a few days; frequently the radium emanation was discharged before the appliances were received by the physicians and of course they were utterly useless.

Still another source of dissatisfaction was the treatment of patients who were apparently incurable, consequently frequent failures occurred. This mistake, if it may be called such, is one to which nearly every surgeon who uses the element subjects himself. It may be said even today that many of us take patients whose cases are forbidding; still the pain from which they suffer, the hemorrhages, discharges and the unpleasant odors, may all disappear under its influence and the patients brought into a more comfortable condition. In fact, those that seemed entirely beyond the reach of remedial agency yield promptly to this remedy. While a cure is out of the question in many of these cases the relief is such that we feel that there is no other means that can in any way equal the beneficial results secured even in these hopeless cases. As shown by this case (fig 1) of lympho sarcoma of the neck, axilla and extensive edema of the thorax, and loss of the use of the arm, any treatment known to us prior to the introduction of radium would have been utterly useless. Of course opium would have relieved the excruciating pain but the patient would have quickly sunk under the disease and succumbed to its ravages. He received ten thousand six hundred milligram hours radiation from nearly a gram of element which caused the tumor to subside nearly one half in size. The pain was so relieved that it was scarcely noticeable, his appetite and strength returned and the outlook was improved. Two months later, he was given a second massive dose. The growth is still regressing and the patient is quite comfortable. We do not expect the disease will entirely disappear but the results achieved so far are such as to warrant the use of the agent in hopeless cases.

While it is too early to make estimates as to the number and extent of the cases improved, it now looks as though by the judicious use of radium and surgery combined twenty-five or thirty per cent. of the hitherto hopeless cases of malignant diseases may be clinically cured. Many of these have remained well after four and five years.

To say that we are surprised at the results of this agent is too weak a word, we are amazed; wide sloughing surfaces even including bone and cartilage are cleaned up and the wound brought into a healthy state and healed. Tumors are dissipated, and the comfort and lives of patients are prolonged far beyond a point possible to be secured by any other treatment. Indeed if radium can achieve no greater success than to lessen pain and other conditions attendant upon the disease it would be sufficient accomplishment.

As stated in a previous article, bad technique has been another fruitful cause of failure. We are gradually correcting these imperfections and many others but a glance at the subject will suffice to convince any that it must necessarily take considerable time to accumulate and classify tables of different forms of disease, methods of treatment sufficiently comprehensive to enable investigators to determine just what cases are suitable for the remedy, just what methods of treatment are best calculated to reach the desired results, just what the dosage should be in each form of the disease, and other questions of vital importance which constantly come before the profession.

With the lapse of years better methods have been developed, accurate instruments of measurement have been invented, the indications for the use of the agent have been narrowed down until one need not often disappoint his patients by holding out claims which have not and cannot be verified. Enough progress has been made to enable us to reach promptly many forms of cancer and sarcoma. In some cases of the maladies a single application causes all symptoms and local manifestations to disappear.

The remedy is gaining quite a foothold in diseases which are by no means malignant; such as goitre, birth marks, benign tumors, especially fibroid of the uterus in which field the most flattering results have been obtained. As Dr. Clark, President of the American College of Surgery, said at the meeting of the American Radium Society in New York City last June, ''Radium is of especial service in the smaller fibroid tumors where infection of the tumor, the appendages or ovarian growths are not present.'' In such cases radium is the treatment of choice, but where the conditions just mentioned are present or the growth is very large and extends above the umbilicus, hysterectomy Dr. Clark believes to be the preferable operation.

The radiologist must have sufficient radium for the curative dose in each individual case and a sufficient volume to enable him to treat with reasonable promptness the number of patients who apply for relief. In superficial epithelioma and rodent ulcer, twenty-five milligrams of radium element will answer for many cases, but in fibroid tumors from fifty to a hundred milligrams will be required and in Hodgkins disease, large sarcoma and the like, a half gram or even more of the element may be demanded.

Five years ago there were only two hospitals in America where sufficient radium was available for the treatment of cancer. There has been vast expansion in the treatment of disease by radium since that time; we now have a National

Fibroid of the Uterus.

Radium Society of about fifty members scattered over the country who are equipped and competent to treat the class of patients afflicted with the various diseases for which radium has now proven itself to be the best if not the only remedy.

Again, most of the medical societies make provisions for the free interchange of thought and discussion of the radium treatment of disease. The medical literature of the country is becoming rich with articles giving technique, dosage and other valuable information. America has already taken a foremost place in the management of cancer and other diseases by the use of radium therapy; and our physicians and surgeons, as shown by their work, rank with the best in this department of practice.

## Figures and Tables

**Figure f1:**
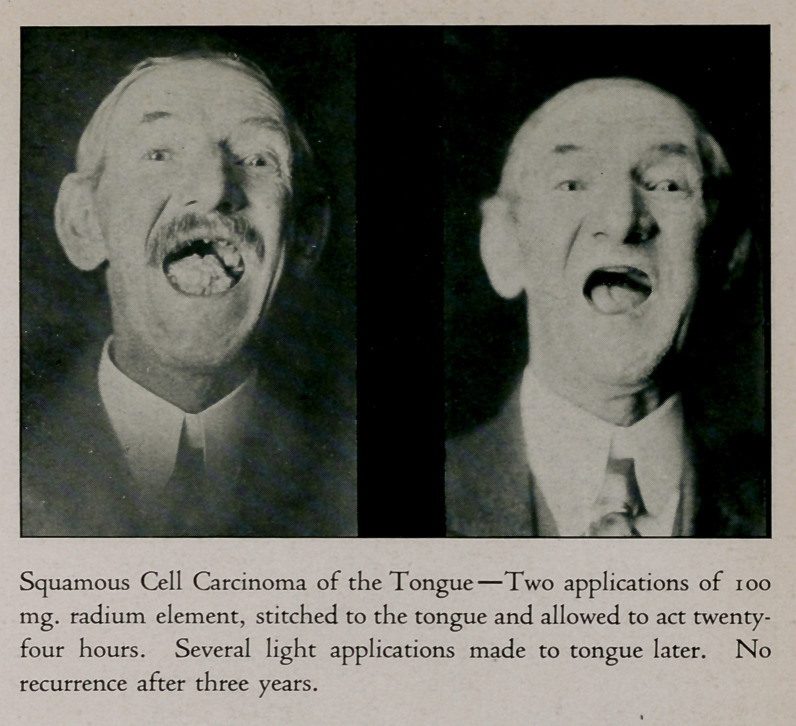


**Figure f2:**
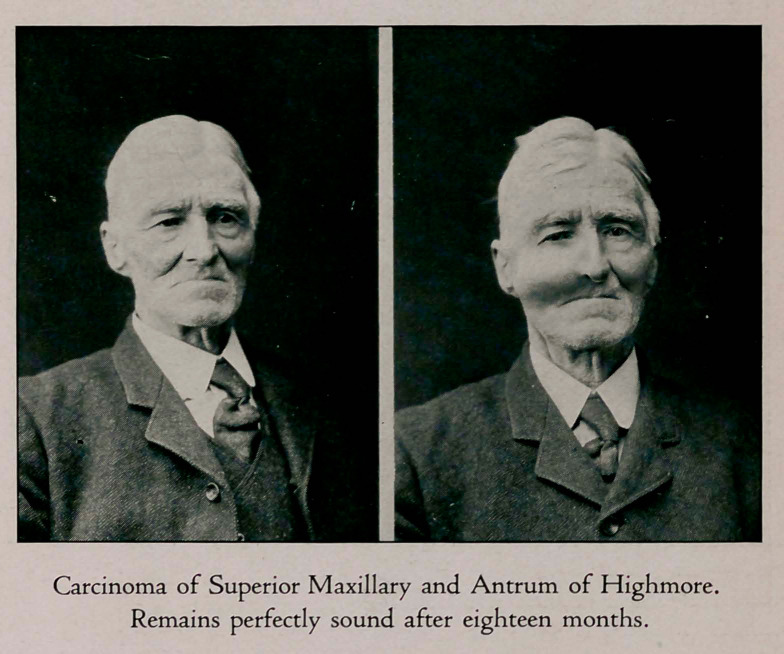


**Figure f3:**
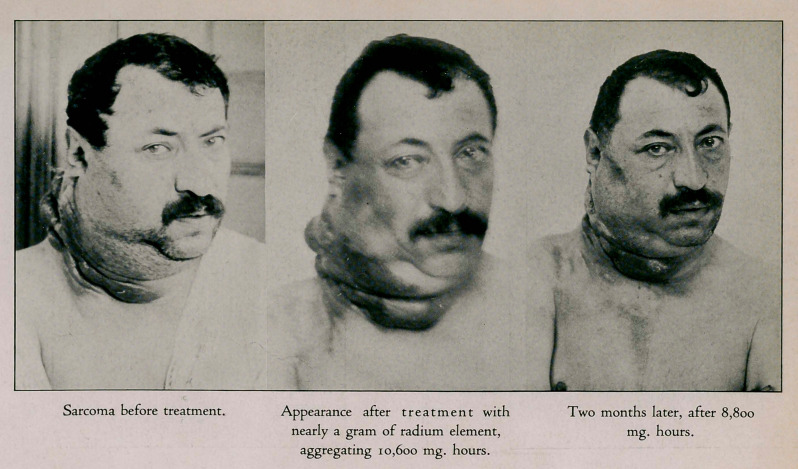


**Figure f4:**